# Dicarboxylic acids counteract the metabolic effects of a Western diet by boosting energy expenditure

**DOI:** 10.1172/JCI181978

**Published:** 2024-06-17

**Authors:** Lidia Castagneto-Gissey, Stefan R. Bornstein, Geltrude Mingrone

**Affiliations:** 1 Department of Surgical Sciences, Sapienza University of Rome, Rome, Italy.; 2 Department of Medicine III, Universitätsklinikum Carl Gustav Carus an der Technischen Universität Dresden, Dresden, Germany.; 3 Division of Diabetes & Nutritional Sciences, School of Cardiovascular and Metabolic Medicine and Sciences, King’s College London, London, United Kingdom.; 4 Università Cattolica del Sacro Cuore, Rome, Italy.; 5 Department of Medical and Surgical Sciences, Fondazione Policlinico Universitario A. Gemelli IRCCS, Rome, Italy.

## Abstract

Obesity has reached pandemic proportion not only in the West but also in other countries around the world; it is now one of the leading causes of death worldwide. A Western diet is rich in saturated fats and provides more calories than necessary, contributing to the rise of the obesity rate. It also promotes the development of liver steatosis, insulin resistance, hyperglycemia, and hyperlipidemia. In this issue of the *JCI*, Goetzman and colleagues describe the effects of consuming dicarboxylic acids (DAs) as an alternative source of dietary fat. The 12-carbon dicarboxylic acid (DC12) was administered to mice at 20% of their daily caloric intake for nine weeks in place of triglycerides. Notably, the change in diet increased the metabolic rate, reduced body fat, reduced liver fat, and improved glucose tolerance. These findings highlight DAs as useful energy nutrients for combatting obesity and treating various metabolic disorders.

## Dicarboxylic acids as the missing fourth nutrient

Medium- and long-chain dicarboxylic acids (DAs) are present in higher plants and animals, deriving from the ω-oxidation of fatty acids (1). In higher plants, DAs are components of natural protective polymers, such as cutin and suberin, a support biopolyester that waterproofs the leaves and fruits, regulating the flow of nutrients and minimizing the harmful impact of pathogens (1). They are β-oxidized in specialized plant peroxisomes, called glyoxysomes, where the glyoxylate cycle, whose intermediates derive from the degradation of reserve or structural lipids, takes place (1).

The paradigm of nutrient composition acknowledges only three energy substrates: glucose, amino acids, and fatty acids. In this issue of the *JCI,* Goetzman et al., (2) confirmed and magnified the evidence of previous investigations establishing the presence and metabolic importance of a fourth nutrient, DAs. While metabolizable DAs are classified as medium-chain fatty acids, they possess two terminal carboxylic groups that permit the formation of dicarboxylic salts, making them easily soluble in water and promptly available as energy. Contrary to typical dietary fatty acids, DAs do not require transport within the circulatory stream, similar to triglycerides, which, as they are insoluble in water, must be incorporated into chylomicrons or lipoproteins such as very-low-density lipoproteins (VLDL) or low-density lipoproteins (LDL). DAs can be immediately β-oxidized, giving rise to acetyl-CoA and succinyl-CoA. Some diseases, such as type 2 diabetes (T2D), result in an inability to rapidly adjust energy substrate utilization and show shortage of glycogen depots in both the liver and skeletal muscle due to impaired glycogen synthesis. Instead, DAs are completely oxidized in T2D in the liver as well as in the muscle and they restore glycogen depots and improve glycemic control (3). Another important characteristic of DAs is their ability to access the mitochondria without the carnitine shuttle, a feature that facilitates complete oxidation (4).

DAs spare glucose utilization and increase glycogen stores in T2D in animal models and in humans (3–9). In fact, 10 g or 23 g of a DA with 10 carbon atoms, such as sebacic acid, added to a 450 kcal meal containing 75 g of glucose but without lipids drastically reduced the plasma glucose peak in people with T2D (3). The circulating levels of insulin in response to the meal were reduced with both amounts of sebacic acid and the rate at which glucose appeared in the circulation after eating was decreased by about 18% (*P* < 0.05) (3).

## DC12 improves insulin resistance and increases energy expenditure

DAs represent a highly performing energy substrate during physical exercise. While metabolically healthy skeletal muscle can switch easily between glucose and fat oxidation in response to homeostatic signals, the skeletal muscle from individuals with T2D or obesity shows a great reduction in this metabolic flexibility (7). Dodecanedioic acid (DC12), a 12-carbon atom straight-chain DA, increases skeletal muscle glycogen stores in people with T2D and might postpone the development of fatigue and increase exercise time (7).

**Figure 1:**
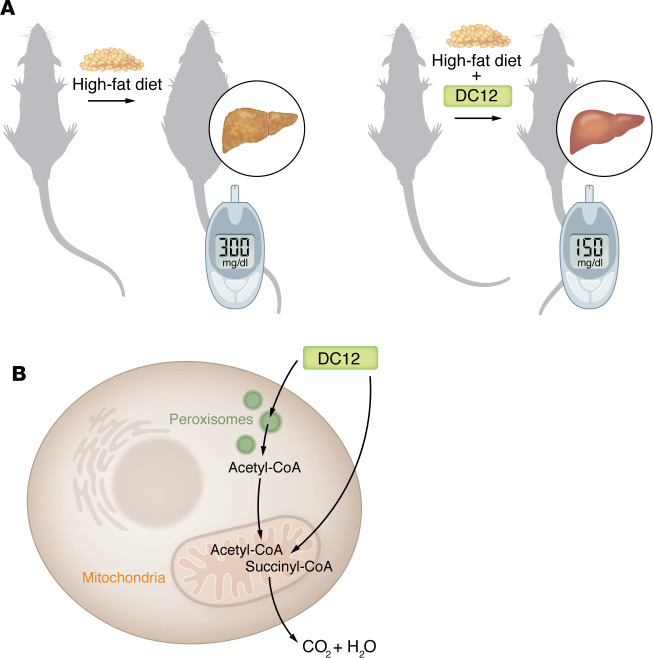
DC12 serves as an alternative source of dietary fat. (**A**) Mice fed a high-fat diet very rich in saturated fatty acids develop obesity and hepatic steatosis and show enhanced circulated levels of glucose and insulin after intraperitoneal glucose administration. In contrast, the addition of 10% DC12 prevents such metabolic disorders. (**B**) DC12 undergoes β-oxidation in the mitochondria and peroxisomes to form the end products acetyl-CoA and succinyl-CoA. While DC12 is completely oxidized to CO_2_ and H_2_O in the mitochondria, acetyl-CoA produced in peroxisomes is transported into the mitochondria where it is completely oxidized.

Goetzman et al., (2) fed mice with diets containing various fat combinations during a nine-week course. Unlike most dietary fats, DAs could not be stored, but, rather, were completely oxidized. Consequently, DA intake did not increase fat mass, despite consumption of 33% of calories as fat (Figure 1). In an era where obesity has become a pandemic (10), DAs might represent a way to reduce the obesity burden in association with antiobesity medications and minimally invasive surgery. Moreover, Goetzman and colleagues (2) demonstrated that DAs, in particular DC12, increased energy expenditure.

Interestingly, Goetzman et al. (2) proved that excess of succinate formation from DA oxidation increased protein succinylation also in white adipose tissue, promoting a process called “beiging” by binding the adipocyte surface through the succinate receptor GPR91. This is a very interesting observation. In fact, it is known that white adipose tissue can undergo the process of browning in response to a variety of stimuli inducing or enhancing the expression of thermogenes that are typically associated with brown fat. White adipose cells become beige or brite cells and are characterized by higher energy expenditure and reduced fat storage (11).

Therefore, a high-fat diet substituted with DC12 increased metabolic rate and prevented obesity in mice, a finding that represents a strategic nutrient approach to slow the progression of the global obesity epidemic. Importantly, mice administered DC12 maintained a normal insulin sensitivity without an increase in blood glucose.

T2D is preceded by a latent period, known as prediabetes, which is caused by peripheral insulin resistance and a compensatory increase in circulating levels of insulin; almost one in three individuals in the United States in 2020 had insulin resistance (12). When pancreatic β cells become unable to compensate the insulin resistance state and insulin secretion fails, overt T2D manifests. A high-fat diet is associated with obesity, whole-body insulin resistance, hyperlipidemia, and glucose intolerance (13). DC12 can overcome such metabolic derangement, preserving the pancreatic insulin secretion machinery and avoiding insulin resistance associated glucose intolerance.

Further, dodecanedioic acid and the shorter DA with 10 carbon atoms, sebacic acid, were proven to improve glucose disposal in humans (3–9).

Another important advantage of dietary DC12 over fatty acids that was highlighted in Goetzman et al. (2) is its storage; DC12 was not stored intracellularly and thus it did not promote fat deposition. In other words, DAs are completely oxidized either in mitochondria or in peroxisomes (Figure 1).

## Dodecanedioic acids counteract the effects of high-fat diet

Supplementation of a 60% high-fat diet with DC12 prevented liver triglyceride accumulation, a feature present in the metabolic-associated fatty liver disease (MAFLD). MAFLD (14) is a pathologic condition, associating hepatic steatosis with T2D and/or obesity and/or metabolic dysregulation (15), with a high prevalence in adults ranging worldwide between 17% and 51% and with the potential to progress to liver cirrhosis and/or hepatocellular carcinoma (15).

## Conclusions and implications

An important question remains open, whether DC12 prevents or reverses liver steatosis alone or if it is also effective in metabolic dysfunction-associated steatohepatitis (MASH). Although the global prevalence of MASH is 5.27% (15), far lower than the prevalence of MAFLD, its clinical picture and evolution are much more severe. Therefore, this topic should be specifically addressed in the future given the scarcity of available therapies (16, 17). It will be critical to perform clinical trials to prove the translational safety and efficacy of DC12.

DAs in general, and DC12 in particular, are safe nutrients that are completely oxidized. They increase energy expenditure without accumulation in adipose tissue, thereby preventing weight gain. Their intake reduces the effects of dietary triglycerides that cause insulin resistance, hyperlipidemia, and liver steatosis. DAs represent the fourth known nutrient — in addition to glucose, amino acids, and fatty acids — known to improve metabolic derangement associated with a high-fat diet, typical of Westernized societies.

## Version 1 06/17/2024

Electronic publication
